
JOURNAL INFORMATION
==============================
NLM Title Abbreviation: ScientificWorldJournal
Journal ID: ScientificWorldJournal
NLM Journal ID: 101131163
Journal ID: TSWJ

The Scientific World Journal

ISSN: 2356-6140
EISSN: 1537-744X
Publisher: Wiley

ARTICLE INFORMATION
==============================
PMCID: PMC5720106
PMID: 21298217
DOI: 10.1100/tsw.2011.27
Article ID: 491356
Article version: 1
Subjects: Clinical Image

Mees' Lines

Mirfazaelian Hadi 1 *
Namazi Mohammad Reza 2
Daneshbod Yahya 3
1 Department of Emergency Medicine, Tehran University of Medical Sciences, Tehran, Iran
2 Shiraz Skin Research Center, Shiraz University of Medical Sciences, Shiraz, Iran
3 Department of Pathology, Dr. Daneshbod Laboratory, Shiraz, Iran
*Hadi Mirfazaelian: hadimir_43@yahoo.comAcademic Editor: Daniel Batlle

Publication date: 2011
Electronic publication date: 2011 Feb 3
Volume: 11
First page: 267
Last page: 268
Received 2010 Nov 16; Revised 2010 Dec 22; Accepted 2010 Dec 23
Copyright: Copyright © 2011 Hadi Mirfazaelian et al.
Copyright year: 2011
License: This is an open access article distributed under the Creative Commons Attribution License, which permits unrestricted use, distribution, and reproduction in any medium, provided the original work is properly cited.
License URL: https://creativecommons.org/licenses/by/3.0/

Keywords: Mees' lines, chemotherapy

==============================
Clinical Image
*TheScientificWorldJOURNAL* (2011) 11, 267–268
ISSN 1537-744X; DOI 10.1100/tsw.2011.27

TheScientificWorldJOURNAL
www.thescientificworld.com

### Mees' Lines

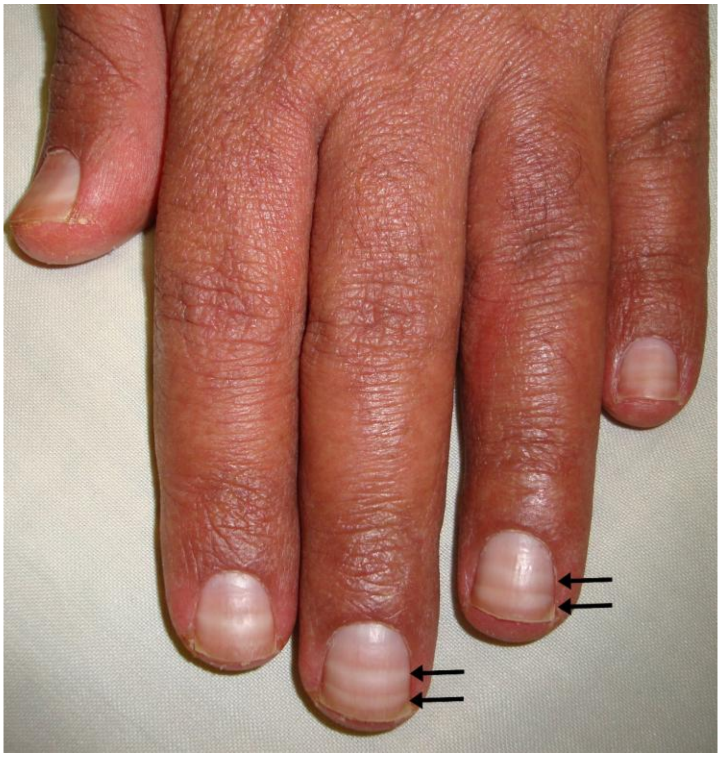

**FIGURE 1.** Mees' lines: white, nonblanching, transverse bands running parallel to the lunula.

Hadi Mirfazaelian[1,*], Mohammad Reza Namazi[2], and Yahya Daneshbod[3]

*[1]Department of Emergency Medicine, Tehran University of Medical Sciences, Tehran, Iran; [2]Shiraz Skin Research Center, Shiraz University of Medical Sciences, Shiraz, Iran; [3]Department of Pathology, Dr. Daneshbod Laboratory, Shiraz Iran*

**E-mail**: Hadimir_43@yahoo.com; namazi_mr@yahoo.com; y@daneshbod.com; daneshbk@yahoo.com

*Received November 16, 2010; Revised December 22, 2010; Accepted December 23, 2010; Published February 3, 2011*

**KEYWORDS**: Mees' lines, chemotherapy

*Corresponding author.
©2011 with author.
Published by TheScientificWorld; www.thescientificworld.com

A 28-year-old man, a known case of acute myelogenous leukemia (AML) (M4), was admitted because of cutaneous infection and pancytopenia 1 month after his second chemotherapy cycle, with no data available about the chemotherapy regimen. On physical examination, in addition to scaling and desquamation on some of his fingers, there were some changes in his nails that started after his first chemotherapy cycle. There were white, nonblanching, transverse bands running parallel to the lunula across the entire nail bed with no palpable ridges (Fig. 1, arrows). These lines, known as Mees' lines (true leukonychia) are related to each chemotherapy cycle, with the distance between them pertaining to the cycles' intervals[1]. Although the patient did not recall the time of the appearance of the lines, knowing that nails grow about 1 mm every 6 to 10 days, the timing of the disease process may be estimated by measuring the distance from the line to the nail bed[2]. They are caused by an insult to the distal nail matrix, which unlike Beau’s lines, do not interrupt growth of the matrix, but causes parakeratosis of the ventral nail plate[3]. Mees' lines can be differentiated from Muehrcke's lines (apparent leukonychia), which are paired white lines caused by vascular congestion in the nail bed, by not fading after digital compression and migration with the growth of the nail in Mees' lines[3]. In addition, thumb involvement is rare in Muehrcke's lines[4]. Differential diagnosis of Mees' lines are arsenic poisoning, Hodgkin’s disease, congestive heart failure, leprosy, malaria, chemotherapy, carbon monoxide poisoning, and other systemic insults[2]. Four months later, the patient underwent bone marrow transplantation, but unfortunately died because of sepsis.

### REFERENCES

1. Park, J. and Li, K. (2010) Images in clinical medicine. Multiple Beau's lines. *N. Engl. J. Med.* **362(20),** e63.
2. Fawcett, R.S., Linford, S., and Stulberg, D.L. (2004) Nail abnormalities: clues to systemic disease. *Am. Fam. Physician* **69(6),** 1417–1424.
3. Huang, T.C. and Chao, T.Y. (2010) Mees lines and Beau lines after chemotherapy. *CMAJ* **182(3),** E149.
4. Morrison-Bryant, M. and Gradon, J.D. (2007) Images in clinical medicine. Muehrcke's lines. *N. Engl. J. Med.* **357(9),** 917.

**This article should be cited as follows:**

Mirfazaelian, H., Namazi, M.R., and Daneshbod, Y. (2010) Mees’ lines. *TheScientificWorldJOURNAL* **11**, 267–268. DOI 10.1100/tsw.2011.27.
